# Electrochemical Aptatoxisensor Responses on Nanocomposites Containing Electro-Deposited Silver Nanoparticles on Poly(Propyleneimine) Dendrimer for the Detection of Microcystin-LR in Freshwater

**DOI:** 10.3390/s16111901

**Published:** 2016-11-11

**Authors:** Mawethu P. Bilibana, Avril R. Williams, Candice Rassie, Christopher E. Sunday, Hlamulo Makelane, Lindsay Wilson, Nomaphelo Ntshongontshi, Abongile N. Jijana, Milua Masikini, Priscilla G. L. Baker, Emmanuel I. Iwuoha

**Affiliations:** 1 SensorLab, Department of Chemistry, University of Western Cape, Robert Sobukwe Road, Bellville, Cape Town 7535, South Africa; m.bilibana@gmail.com (M.P.B.); crassie@uwc.ac.za (C.R.); csunday@uwc.ac.za (C.E.S.); hlamzam@gmail.com (H.M.); 2724554@myuwc.ac.za (L.W.); 2917310@myuwc.ac.za (N.N.); 2505749@myuwc.ac.za (A.N.J.); mmasikini@uwc.ac.za (M.M.); pbaker@uwc.ac.za (P.G.L.B.); 2 Department of Biological and Chemical Sciences, The University of the West Indies, Cave Hill, St. Michael BB11000, Barbados; avril.williams@cavehill.uwi.edu

**Keywords:** aptamer, aptatoxisensor, cyanotoxins, electrochemical biosensor, metallodendrimer, microcystin-LR, nanosensor

## Abstract

A sensitive and reagentless electrochemical aptatoxisensor was developed on cobalt (II) salicylaldiimine metallodendrimer (SDD–Co(II)) doped with electro-synthesized silver nanoparticles (AgNPs) for microcystin-LR (L, l-leucine; R, l-arginine), or MC-LR, detection in the nanomolar range. The GCE|SDD–Co(II)|AgNPs aptatoxisensor was fabricated with 5’ thiolated aptamer through self-assembly on the modified surface of the glassy carbon electrode (GCE) and the electronic response was measured using cyclic voltammetry (CV). Specific binding of MC-LR with the aptamer on GCE|SDD–Co(II)|AgNPs aptatoxisensor caused the formation of a complex that resulted in steric hindrance and electrostatic repulsion culminating in variation of the corresponding peak current of the electrochemical probe. The aptatoxisensor showed a linear response for MC-LR between 0.1 and 1.1 µg·L^−1^ and the calculated limit of detection (LOD) was 0.04 µg·L^−1^. In the detection of MC-LR in water samples, the aptatoxisensor proved to be highly sensitive and stable, performed well in the presence of interfering analog and was comparable to the conventional analytical techniques. The results demonstrate that the constructed MC-LR aptatoxisensor is a suitable device for routine quantification of MC-LR in freshwater and environmental samples.

## 1. Introduction

There is current interest in algal toxins, which are produced by fresh water blue-green algae (commonly known as cyanobacteria), because of the threat they pose to freshwater ecosystems, reservoir drinking water, irrigation and recreation (e.g., swimming pools, and hot tubs) systems [1,2,3]. The major menace associated with cyanobacteria lies in their ability to produce potent toxins (cyanotoxins) and secondary metabolites such as microcystins (MCs) [4]. Hence, the detection and monitoring of water contaminated with these toxic compounds are necessary for the safety and security of humans and animals. The World Health Organization (WHO) guidelines [5,6,7,8,9] have detailed values of 1 μg·L^−1^ per toxin in drinking water and a Tolerable Daily Intake (TDI) of 0.04 μg·kg^−1^ body weight per day in relation to basic human health and safety. With these guidelines in place, several screening procedures for microcystin-LR (L, l-leucine; R, l-arginine), or MC-LR, have been widely reported in literature; among these are enzyme-linked immunosorbent assay (ELISA), immunosensing, high performance liquid chromatography (HPLC) and bioassay. However, these procedures are generally time-consuming as they require several steps inclusive of sample pre-treatment which requires skilled personnel. In response to these limitations, electrochemical aptamer-based toxin biosensors (aptatoxisensor) have been established to advance the screening of MC-LR in water [10,11]. Aptamers are artificial oligonucleotide receptors that have highly discriminative affinities for their targets which range from small molecules to large macromolecules such as proteins and even cells [12]. In the electrochemical biosensor field, highly sensitive and stable biosensors are widely appreciated since they are able to detect different analytes. Therefore, artificial oligonucleotide receptors, such as aptamers, are widely recognized as promising tools for environmental toxin screening, as they are thermodynamically stable, easily synthesized and offer significant flexibility in creating sensitive biosensors for the screening of toxins [13]. In 2012, Singh et al. reported on the current trends associated with the development of biosensors for MCs detection including enzyme-based biosensors, immunosensors and nucleic acid biosensors amongst others [14]. In regards to quality water management, the authors suggested that a nucleic acid biosensor could fulfill the demand for the efficient screening of MC-LR when compared to the other types of biosensors. Consequently, Ng et al. reported the selection and characterization of high affinity congener-specific MC-targeting DNA-based aptamers [15]. Furthermore, these aptamers were applied as part of an electrochemical aptatoxisensor, to detect congener-specific MCs, resulting in a high response sensitivity with a detection limit (LOD) of 10 pg·L^−1^. In 2013, Lin et al. reported an electrochemical impedance label-free aptatoxisensor, where DNA aptamers were immobilized on gold electrode for the screening of MC-LR in water with a LOD of 0.018 ng·L^−1^ [16]. Then, in 2015, Wang et al. reported an aptamer-based biosensor for the colorimetric detection of MC-LR—with an LOD of 0.05 ng·L^−1^—supported on the disassembly of orient-aggregated gold nanoparticle dimers [17]. Recently, a report by Du et al. demonstrated an MC-LR aptatoxisensor with a LOD of 0.03 pg·L^−1^ and an analytical recovery greater than 97.8% [18]. In order to increase the electrical signal response of this aptatoxisensor, the DNA aptamer sequence was immobilized on a surface of BiOBr nanoflakes/N-doped graphene nanocomposites onto Indium tin oxide (ITO) electrode.

In recent times, studies have shown that dendrimers are a structural well-ordered class of polymers [19]. Predominantly, dendrimers consist of a core, self-replicating branching units and peripheral surface groups. The dendrimers can be wholly modified with functional groups at different positions. A body of literature [20,21,22,23,24] has reported metallo-dendrimers where the dendrimeric structure is connected to transition metal complexes, and where the metal ion is bonded to two different molecules at the periphery of the dendrimer. The redox reactions of the transition-metal complexes, in combination with the organic structure of the dendrimer, contribute to the organization of the molecular electronic communication in the nanomaterial. Indeed, the symmetry, additional functional groups, internal cavities and nanosize structure make these nanocomposites ideal candidates for conductive platforms that can assist in the transport of electrons thereby enhancing the sensitivity of biosensors. Hence, various research groups have demonstrated dendrimer functionalized electrochemical aptatoxisensors [20,21]. Others have extensively demonstrated that first generation electroactive iron, cobalt, nickel and copper poly(propyleneimine) metallodendrimers as viable candidates for biosensing as the diffusion coefficient is not affected by oxygen [22,23]. Moreover, the inclusion of noble metal nanoparticles onto the surface of dendritic structures has provided access to novel conductive materials capable of facilitating direct electron transfer and retaining good bioactivity [24,25,26]. Silver nanoparticles (AgNPs) with unique quantum characteristics, large specific surface area and the ability to quickly transfer photoinduced electrons at the surfaces of colloidal particles have been reported [27,28,29]. In 2014, Kavosi et al. [30] reported a biosensor where gold nanoparticles on a poly(amidoamine) (PAMAM) dendrimer were loaded onto the multi-walled carbon nanotubes (MWCNTS)/chitosan/ionic nanocomposite, while Baccarin et al. [31] demonstrated silver nanoparticles encapsulated within a poly(amidoamine) dendrimer nanocomposite. Both biosensors exhibited greatly enhanced direct electron transfer reactions between redox enzymes and the electrode surface.

**Figure 1:**
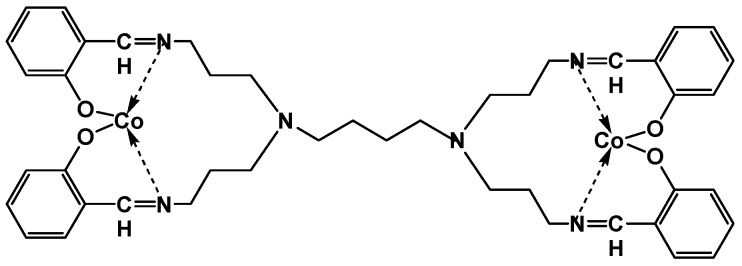
First-generation cobalt(II) salicylaldiimine metallodendrimer (SDD–Co(II)).

In this present work, an aptatoxisensor was fabricated using a glassy carbon electrode (GCE) with a 5′ thiolated DNA aptamer which was self-assembled on a nanocomposite composed of electro-synthesized AgNPs on the surface of cobalt(II) salicylaldiimine metallodendrimer (SDD–Co(II)) (Figure 1). The resulting aptatoxisensor was characterized by cyclic voltammetry (CV) and was used to screen for trace amounts of microcystin-LR (MC-LR). The potential of the aptatoxisensor for application in the detection of MC-LR in freshwater samples was also investigated.

## 2. Experimental

### 2.1. Materials and Methods

Reagent grade disodium hydrogen phosphate (98%), 2-mercaptoethanol, potassium dihydrogen phosphate (99%), 2,5-dimethoxyaniline, poly(4-styrenesulfonic acid), sodium hydrogen phosphate, potassium dihydrogen phosphate, hydrochloric acid, potassium chloride, solutions of Microcystin-LR (L, l-leucine; R, l-arginine) (MC-LR), Microcystin-YR (Y, l-tyrosine; R, l-arginine) (MC-YR) and Microcystin-RR (l-arginine; l-arginine) (MC-RR), silver nitrate (AgNO_3_) and trizma hydrochloride (Tris-HCl) were obtained from Sigma Aldrich, South Africa and used without any further treatment. Cobalt(II) salicylaldiimine metallodendrimer was synthesized using the method from literature [30]. The DNA aptamer-MC-LR sequence was reported in the literature by Ng et al. [15]. (MCLRA):5′-SH(CH_2_)6’GGCGCCAAACAGGACCACCATGACAATTACCCATACCACCTCATTATGCCCCATCTCCGC-3′ was purchased from Inqaba biotech (Johannesburg, South Africa). The DNA aptamer solution was prepared by dissolving DNA in the binding buffer (50 mM Tris-HCl, pH 7.5, 150 mM NaCl, 2 mM MgCl_2_) and denatured by heating to 95 °C for 5 min, and then cooling gradually in ice to room temperature, in order to obtain the inherent 3-D structure. The stock solution of MCs was diluted using phosphate buffer solution to obtain appropriate working concentrations. All solutions were prepared using deionized water (18.2 MΩ·cm) purified by a Milli-Q^TM^ system (Millipore). Analytical grade argon gas was purchased from the Afrox Company, South Africa. Alumina polishing pads and powder (0.05, 0.3 and 1.0 μm) were obtained from Buehler, Illinois, USA. Phosphate buffer solution (PBS) (0.1 M, pH 7.4) containing 0.1 M KCl was used as the supporting electrolyte. All experiments were carried out at room temperature unless stated otherwise.

### 2.2. Apparatus

Cyclic voltammetry (CV) investigations were performed with a computer-controlled BioAnalytical Systems (BAS) 100 W integrated automated electrochemical workstation (BAS, West Lafayette, IN, USA). The classic three-electrode system was adopted as the cell system with a modified glassy carbon electrode (GCE) as the working electrode, a platinum wire as the counter electrode, and Ag/AgCl as the reference electrode. Fourier transform infrared spectra were acquired using a Fourier transform infrared (FTIR) spectrometer (PerkinElmer Spectrum 100, PerkinElmer Incorporated, Shelton, CT, USA).

### 2.3. Preparation of GCE|SDD–Co(II)|AgNPs

Prior to the electro-synthesis of AgNPs on GCE|SDD–Co(II) the working electrode surfaces of GCE were polished using alumina (1.0, 0.3, and 0.05 μm) slurry (Al_2_O_3_), ultrasonicated for 5 min in ethanol and water, respectively, then dried in a nitrogen stream. A thin film of 5 mM cobalt(II) metallodendrimer (SDD–Co(II)), prepared using 1:1 (*v*/*v*) acetone-ethanol solution was deposited onto the surface of the clean GCE and left to dry for 1 h to allow for physical adsorption to occur. The surface of the electrode was lightly rinsed with ethanol to remove any unbound salicylaldiimine dendrimer and dried under a stream of nitrogen gas. The resulting electrode was denoted GCE|SDD–Co(II). For the electro-synthesis of silver nanoparticles on the GCE|SDD–Co(II), the electrode was placed in a phosphate buffer solution containing 3 mM AgNO_3_, which was then cycled five times within a window potential of −400 to 800 mV using cyclic voltammetry. During the electrodeposition process, AgNPs attached onto the GCE|SDD–Co(II) film. The cyclic voltammetric electrochemical behavior of GCE|SDD–Co(II)|AgNPs was studied in 0.1 M phosphate buffer pH 7.4 in the potential range of −400 to 800 mV. Electrochemical impedance spectroscopy (EIS) was performed from 10^5^ to 10^−1^ Hz in a 7.4 pH 0.1 M phosphate buffer pH 7.4. All electrochemical experiments were conducted under an inert atmosphere obtained by degassing the cell with argon for approximately 10 min.

### 2.4. Aptatoxisensor (GCE|SDD-Co(II)|AgNPs|MCLRA) Development and MC-LR Detection

**Figure 2:**
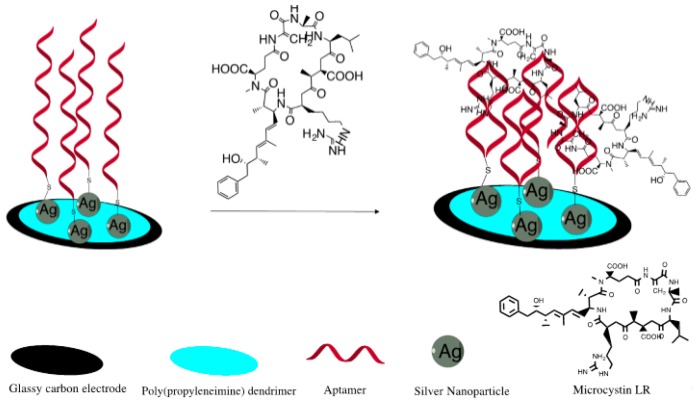
Schematic representation of cobalt(II) salicylaldiimine metallodendrimer aptatoxisensor preparation.

Five microliters of 1 µM MC-LR 5′-thiol ssDNA aptamer (MCLRA) was suspended in a binding buffer containing 50 mM Tris-HCl (pH 7.5), 150 mM NaCl and 2 mM MgCl_2_. The suspension was coated onto the GCE|SDD–Co(II)|AgNPs surface and then the electrode was immediately capped with a pipetting tip in order to allow the immobilization of the DNA aptamer to occur in a solvent-saturated micro-atmosphere for 24 h at 4 °C. The principle of development of the apatatoxisensor is demonstrated in Figure 2. The resulting aptatoxisensor (GCE|SDD-Co(II)|AgNPs|MCLRA) was then incubated for 1 h in 0.1 M phosphate buffer (pH 7.0) containing 1 mM 6-mercapto-1-hexanol (MCH) to remove unbound oligonucleotides and was subsequently rinsed with binding buffer. The voltammetric measurements of the aptatoxisensor were performed in a 5 mL cell containing 0.1 M PBS (pH 7.4) in a potential range of −400 to 800 mV (vs. Ag/AgCl) at a scan rate of 40 mV·s^−1^. The CV responses of the GCE|SDD–Co(II)|AgNPs|MCLRA aptatoxisensor were measured at room temperature by incubating the aptatoxisensor electrode for 1 min, in varying concentrations of MC-LR (0.1–5.0 µg·L^−1^).

## 3. Results and Discussion

### 3.1. Characterization of GCE|SDD–Co(II)|AgNPs

**Figure 3:**
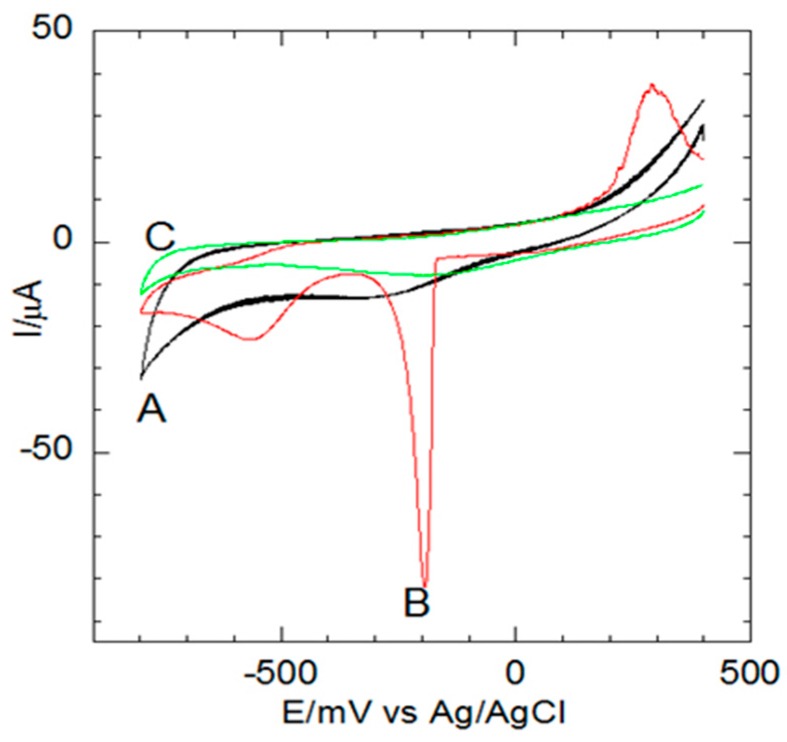
Cyclic voltammetric traces for: (**A**) GCE|SDD–Co(II)|(black); (**B**) GCE|SDD–Co(II)|AgNPs|(red); and (**C**) GCE|SDD–Co(II)|AgNPs|MCLRA (green) in 0.1 M PBS at a 100 mV·s^−1^ scan rate.

Modification of electrode surfaces with multi-layered nanocomposites is a widespread technique used in the fabrication of high performance reagentless biosensors and characterization of the amended surfaces is integral. Hence, Figure 3 shows the voltammograms of (A) GCE|SDD–Co(II)|(black), (B) GCE|SDD–Co(II)|AgNPs|(red) and (C) GCE|SDD–Co(II)|AgNPs|MCLRA (green) both of which demonstrated good electroactivity in PBS (pH 7.4) within the potential range commonly found in studies of direct electron transfer of DNA aptamers. A formal potential (*E’*) value of close to 80 mV was obtained for the GCE|SDD–Co(II)|AgNPs|MCLRA electrode which is an indication of electron transfer (voltammogram C). This voltammetric response implies that the nanocomposite has provided a suitable micro-environment for the immobilized DNA aptamer, resulting in altered (increased) conductance through the device [32].

Indeed, integration of nanocomposites into the electrode allows for the effective modification of the electrode surface such that the DNA aptamer is localized close to the electrochemical interface thereby minimizing interferences which may lead to undesired side reactions and large background currents. Figure 3A revealed that the AgNO_3_ was successfully reduced to AgNPs, through CV, without any reducing agents, which decorated the GCE|SDD–Co(II) nanocomposite. In Figure 3A, the redox peaks, attributed to the AgNPs, are clearly evident in the voltammograms for the GCE|SDD–Co(II)|AgNPs electrode at −90 mV (vs. Ag/AgCl). This activity indicates a strong interaction between the dendrimer and the electrodeposited AgNPs resulting in sufficient electron transfer between the DNA aptamers and the GCE|SDD–Co(II)|AgNPs surface as a result of the latter providing the MC-LR aptamers with effective adsorption sites so that the active centers can be easily accessed [31].

### 3.2. EIS Characterization of GCE|SDD–Co(II)|MCLRA and GCE|SDD–Co(II)|AgNPs

**Figure 4:**
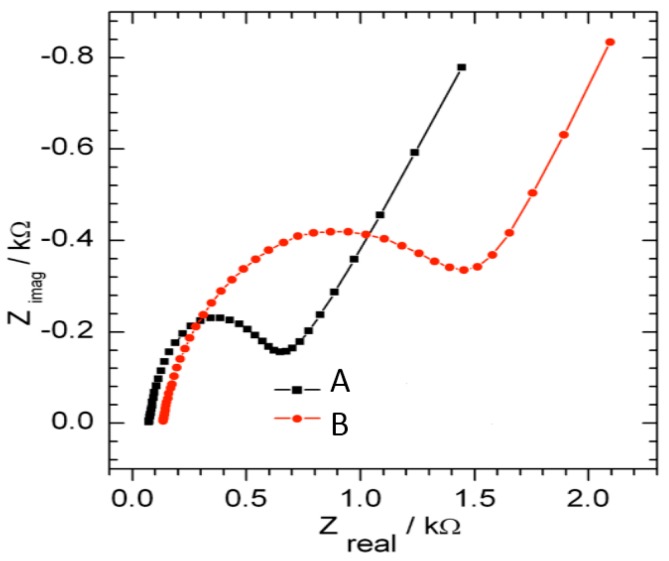
EIS Nyquist plots of the modified electrodes: (**A**) GCE|SDD–Co(II)|AgNPs; and (**B**) GCE|SDD–Co(II)|AgNPs|Aptamer, for experiments performed in 0.1 M PBS (pH 7.4).

EIS was used to characterize each step of the biosensor fabrication. The EIS measurements indicated that better aptatoxisensor characteristics were obtained after an applied *E’* of +80 mV. The electron transfer resistance (R_et_) was proportional to semicircular diameter at high frequencies [32]. The different Nyquist diagrams obtained in the presence of the grafted GCE|SDD–Co(II)|AgNPs (A) and GCE|SDD–Co(II)|AgNPs|Aptamer layer (B) were modeled according to Randles circuit (Figure 4), using Zplot/Zview software. From the figure, the Nyquist plot of GCE|SDD–Co(II)|AgNPs revealed a R_et_ of 500 Ω. The electro-synthesised deposition of AgNPs, resulted in a sharply reduced charge transfer resistance (R_et_) for the electrode which could be attributed to the well-known conducting ability of AgNPs. The amount of AgNPs deposited on the electrode surface increased the nanocomposite surface area and owing to the large resistance of the aptamer, the R_et_ of GCE|SDD-Co(II)|AgNPs dramatically increased to 1.50 KΩ after grafting of the aptamer. This is not surprising given that there is strong electrostatic repulsion of the redox probe by densely grafted aptamers. Each assembly step characterized by EIS afforded results consistent with the corresponding CV data. The GCE|SDD–Co(II)|AgNPs|Aptamer nanocomposite enhanced the electronic transmission capacity and increased the amount of immobilized probe. Therefore, the sensitivity and stability of the aptatoxisensor is promoted by the GCE|SDD–Co(II)|AgNPs|nanomaterial.

### 3.3. FTIR Structural Characterization of GCE|SDD–Co(II)|AgNPs

**Figure 5:**
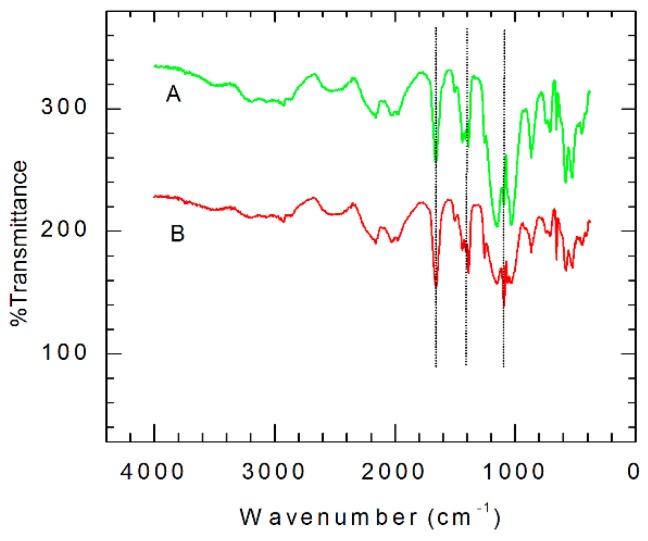
FTIR of: (**A**) GCE|SDD–Co(II); and (**B**) GCE|SDD–Co(II)|AgNPs.

The FTIR spectra in Figure 5 verified the incorporation of AgNPs into the Co(II) salicylaldiimine metallodendrimer (GCE|SDD–Co(II)). The coordination of the SDD to the cobalt ion, through the amine nitrogen atoms, is revealed by the C−N stretching band at 1636 cm^−1^ in the spectrum of the cobalt Schiff base electrode, GCE|SDD–Co(II). In addition, the presence of the band at 1528 cm^−1^ in the spectrum of the GCE|SDD–Co(II) (Figure 5A) implies that there is complex formation. The band at 528 cm^−1^ is representative of the Co–O stretching frequency. The spectrum of GCE|SDD–Co(II)|AgNPs (Figure 5B) shows a shift in the C–O stretching vibration of the phenoxy group from the region 1148–1161 cm^−1^ to higher frequency indicating AgNPs–O organization. These shifts, brought about by the absorption of silver, show that the silver nanoparticles are bound and incorporated into the GCE|SDD–Co(II) nanocomposite.

### 3.4. Electrochemical Determination of MC-LR

The thiolated MC-LR aptamers form a self-assembled monolayer on the surface of the GCE|SDD–Co(II) nanocomposite through the−SH covalent attachment to the AgNPs [33] which also mediate the electrical properties induced by the binding of the MC-LR to the DNA aptamer. However, the DNA aptamer oligonucleotides may not attach to the surface exclusively through the sulfur atom of the thiol group as nitrogen-containing nucleotide side chains can and may also interact directly with the AgNPs on the GCE|SDD–Co(II)|AgNPs surface. To diminish such non-specific binding of the DNA aptamer to the AgNPs on the surface of GCE|SDD–Co(II)|AgNPs, 6-mercapto-1-hexanol (MCH) was used [34]. As a result, only thiolated DNA aptamers are adsorbed onto the surface of the modified GCE|SDD–Co(II)|AgNPs through the thiol sulfur atom as nonspecifically bound DNA aptamers are removed by the MCH treatment [34,35].

**Figure 6:**
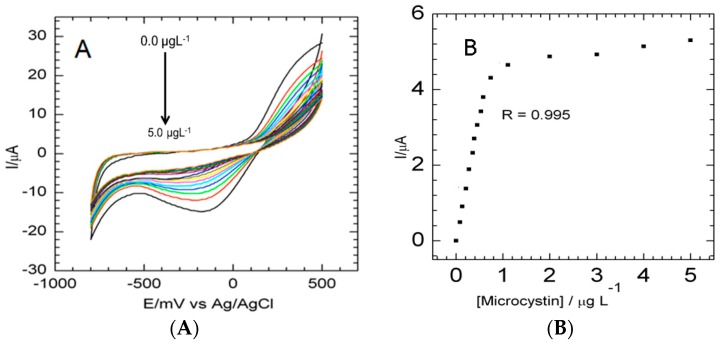
(**A**) Cyclic voltammograms of aptatoxisensor for detection of MC-LR; and (**B**) calibration curve showing GCE|SDD–Co(II)|AgNPs|MC-LRA responses to MC-LR.

A series of voltammetric responses was used to measure the interaction between MC-LR and the thiolated aptamer on the surface of GCE|SDD–Co(II)|AgNPs in 0.1 M PBS in the absence and presence of 0.1 µg·L^−1^ MC-LR at a scan rate of 40 mV·s^−1^. After every aliquot of MC-LR, the 0.1 M PBS (pH 7.4) solution was stirred for 30 s then purged for 1 min and the current signals were recorded. In Figure 6A, the voltammograms indicate the complex formation between the aptamer and the MC-LR molecules on the surface of GCE|SDD–Co(II)|AgNPs where it can be seen that the current decreases after the MC-LR treatment, based on the specific complexation of MC-LR to the aptamer. With the addition of the MC-LR, the conformation of the DNA aptamer changes to a locked structure and so the interface of the biosensor changes due to interfacial kinetics of electron transfer. The effect on the electron transfer at the interface of GCE|SDD–Co(II)|AgNPs|MCLRA is predominantly the result of steric hindrance and electrostatic repulsion owing to the complex formation which concludes in increased mass transfer resistance of the electrochemical probe and hence a decrease in cathodic and anodic currents [36,37]. The decrease in the peak currents and the shifting of the peak potentials illustrate with certainty that the aptamer-MC-LR complex formed. In Figure 6B, the calibration curve was found to be linear within a concentration range of 0.1–1.1 µg·L^−1^ with a correlation coefficient of 0.995 and a calculated limit of detection (LOD), for the first oxidation peak, of 0.04 µg·L^−1^ (*n* = 3). LODs were calculated based on the standard deviation (SD) of the current response and the slope of the calibration curve (S) according to the formula: LOD = 3.3 (SD/S). Serial dilution tests of MC-LR revealed that even 0.01 µg·L^−1^ MC-LR was measureable if a lower concentration of aptamer was immobilized on the nanocomposite platform. This high sensitivity of the aptatoxisensor may be accredited to the silver nanoparticles and the nanocomposite on the surface of the GCE, which significantly improved the loading of the DNA aptamer probe and hence evidently improved the sensitivity for the MC-LR.

### 3.5. Application of Aptatoxisensor for Real Sample Aanalysis

To establish the performance of the aptatoxisensor in real-world water analysis, we investigated its response to tap, distilled, and wastewater samples. There is still a pressing need to develop new strategies that couple pg·L^−1^ with the concentration (i.e., 0.1 µg·L^−1^) usually found in the contaminated water sites. The wastewater sample was obtained from the City of Cape Town scientific services. The recoveries were evaluated for the wastewater, tap and distilled water using standard spiking methods where solutions of the three samples were spiked with MC-LR standard solutions of three different concentrations. Table 1 shows the recovery results of MC-LR after the three water samples were spiked with 0.01, 0.02 and 0.04 µg·L^−1^ concentrations. The average recoveries range from 94% to 115% with relative standard deviations (RSD) less than 5% based on triplicate tests at each concentration.

**Figure 7:**
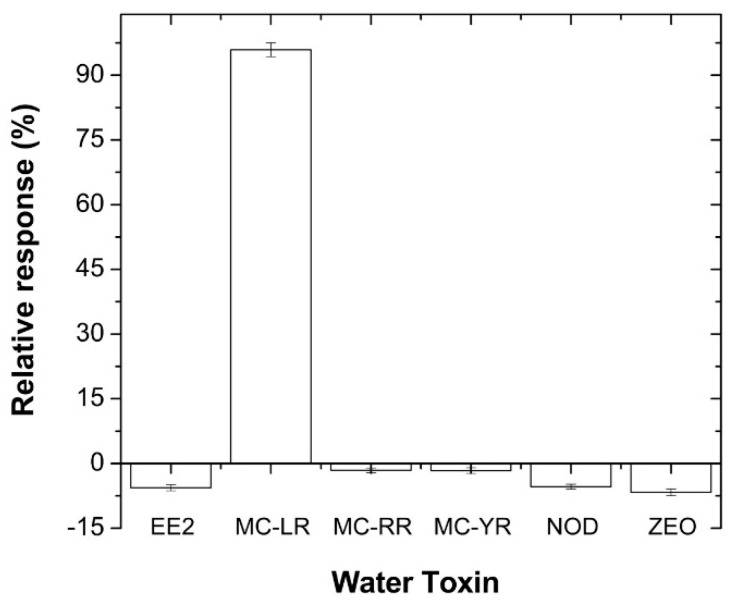
Relative CV responses used to evaluate cross-reactivity between freshwater toxins. EE2 = 17β-estradiol; NOD = Nodularin-R; ZEO = zearalenone.

Generally, in water algal bloom, MC-LR may coexist with some algal toxins such as Nodularin-R (NODLR), MC-RR, MC-YR, 17β-estradiol (EE2), as well as some food toxins such as zearalenone (ZEO) [38,39,40,41]. Therefore, there is a need to evaluate the cross-reactivity of the aptatoxisensor. In order to realize this objective, the voltammetric peak currents were recorded for 0.1 µg·L^−1^ solutions of the abovementioned toxins in 5 mL PBS solution. The relative responses of the peak currents were evaluated from (*E*_p_–*E*_0_)/*E*_0_, where *E*_0_ and *E*_p_ refer to the peak current value obtained before and after incubation, respectively, with the different MC-LR concentration. Figure 7 demonstrates the cross-reactivity effect on the aptatoxisensor when it was applied to a PBS solution spiked with 0.1 µg·L^−1^ concentrations of different algal toxins that are usually present in water. These results showed that the aptatoxisensor was very efficient at distinguishing between not only MC-LR and other toxins, but also between MC congeners differing by only one amino acid residue—leucine for MC-LR, arginine for MC-RR and tyrosine for MC-YR [42]. Hence, the alkyl chain of leucine (LR) present in MC-LR seems to have a greater affinity for the aptatoxisensor than the heteroatom chain of MC-RR and the aromatic ring of tyrosine of MC-YR.

Therefore, the approach applied in this study proves that the developed aptatoxisensor is rapid and hence cost-effective as the whole procedure of detection and quantification takes no more than 1 min, which is time-saving compared to conventional methods.

### 3.6. Comparison of Aptatoxisensor with Traditional/Existing Analytical Procedures

To date, there are three commercial microplate ELISA kits available for MC detection and quantification with LODs of 0.1 μg·L^−1^. The commercial microplate ELISA kits have working ranges between 0.1 and 5 μg·L^−1^, which is appropriate for water monitoring as the WHO guidelines detail a limit of detection of 1 μg·L^−1^ per toxin in drinking water. However, ELISA and liquid chromatography (LC) methods are expensive and require trained personnel, and, in the latter case, expensive instrumentation. In addition, the commercialized ELISA kits have high cross-reactivity with MCs congeners. Alternatively, there is literature, albeit minimal, that has reported on the production of aptatoxisensors for MCs. They, however, have not been proven suitable for onsite freshwater toxins analysis [15,16,43] but have shown that aptatoxisensors can be used as sensing systems for MCs.

Herein, we have demonstrated an aptatoxisensor that not only has high sensitivity but is also rapidly fabricated using an inexpensive approach. Indeed, the aptatoxisensor is highly specific and easy-to-use for on-site analysis of fresh/waste water. Generally, we desire that aptatoxisensors have low LODs, in the picogram per liter range, and a very low cross-reactivity with other antigens. In this study, the aptatoxisensor returned a working range of 0.1–1.1 μg·L^−1^ with LOD of 0.04 μg·L^−1^ and was successful at distinguishing between the MC congeners.

Further, the aptatoxisensor was validated with tap, distilled and wastewater samples spiked with 0.05 µg·L^−1^ concentrations MC-LR. The spiked water samples were analyzed using ELISA and CV and the results are collected in Table 2. The aptatoxisensor was able to detect the MC-LR concentration in tap and wastewater while no MC-LR was detected when the ELISA method was used. As such, the proposed method should be applicable for on-site analysis to screen trace amounts of MCs in water samples.

The LOD of the aptatoxisensor used in this study was compared with commercially available ELISA kits and other analytical methods that are traditionally utilized in the screening of MCs in water samples (Table 3). We admit that conventional techniques are ideal for the analysis of MCs, however for daily analyses and emergency monitoring, aptatoxisensors are valuable tools considering their specificity and affinity for MC-LR, good linear range and low LOD. The enzyme-based biosensors are disadvantageous when compared with the aptatoxisensor, as the recognition enzyme at the center of the biosensor activity usually becomes inhibited by contaminants present in the water sample and so returns in faulty results. In addition, these biosensors are not usually efficient at discriminating between the various cyanotoxin analogs (e.g., nodularins and MC-LR) present in the same sample [44,45,46,47]. The novel aptatoxisensor presented in this study does not suffer from such limitations.

Finally, the stability of the aptatoxisensor was investigated by examining its response, after eight weeks of storage in binding buffer at 4 °C. When the response of the stored aptatoxisensor was compared to freshly prepared aptatoxisensor, it was found that 93.5% of the current response was retained, suggesting that the aptatoxisensor showed good stability.

## 4. Conclusions

A simple and rapid MC-LR aptatoxisensor has been successfully developed by immobilizing a thiolated aptamer on a surface of a GCE|SDD–Co(II) dendrimeric nanocomposite. Silver nanoparticles were successfully incorporated onto the modified GCE cobalt(II) salicylaldiimine metallodendrimer through an electro-synthetic approach using a CV method without reducing and stabilizing agents. The GCE|SDD–Co(II)|AgNPs nanocomposite acted as an interface to immobilize the thiolated DNA aptamer using the relatively uncomplicated and inexpensive method of thiol self-assembled monolayers (SAMs) on AgNPs and allowed for the detection of MC-LR in freshwater samples. The voltammograms obtained from the GCE|SDD–Co(II)|AgNPs|thiolated aptatoxisensor exhibited a linear range of 0.1–1.1 μg·L^−1^ with a LOD of 0.04 μg L^−1^. Moreover, this aptatoxisensor was immune to any cross-reactivity effect with other environmental toxins, enabling the developed aptatoxisensor to be applied in the field analysis of freshwater samples with trace amounts of MCs. Confirmation studies validated that the data produced by the aptatoxisensor were comparable to those generated by the conventional screening methods for MC-LR, which are utilized by the U.S. Environmental Protection Agency (EPA), WHO and the South African Department of Water Affairs, amongst others.

## Figures and Tables

**Table 1:** Real sample aptatoxisensor analysis.

Sample	[MC-LR] Added (µg·L^−1^)	[MC-LR] Detected (µg·L^−1^)	Recovery (%)	RSD (%)
Distilled water	0	ND	-	-
	0.01	0.0095	95.0	4.40
	0.02	0.0195	97.3	1.50
	0.04	0.0394	98.4	1.06
Tap water	0	0.0047	-	-
	0.01	0.0096	94.3	5.01
	0.02	0.0194	98.7	1.78
	0.04	0.0395	97.6	0.96
Wastewater	0	0.0068	-	-
	0.01	0.0109	109.0	1.52
	0.02	0.0290	104.2	1.18
	0.04	0.0460	115.0	5.06

(-): Undefined values; 0: no spiking with MC-LR; ND: Not detected.

**Table 2:** Analysis of MC-LR content in spiked (0.05 µg·L^−1^) tap, distilled and waste water samples.

Real Samples	Elisa Result (µg·L^−1^)	Aptatoxisensor Result (µg·L^−1^)
Tap water	ND	0.047
Distilled water	ND	ND
Wastewater	ND	0.078

ND: Not detected.

**Table 3:** Comparison of the aptatoxisensor with similar electrochemical sensors and selected analytical techniques used in the screening of MC-LR

Techniques	DLR (µg·L^−1^)	LOD (µg·L^−1^)	References
MALDI-TOF MS	0.11–5.0	0.015	[48]
Liquid chromatography	10–500	0.1	[49]
CLEIA	0.062–0.65	0.032	[50]
Mediated label-free Au/AuNPs amperometric immunosensor	0.05–15	0.02	[51]
MWCNT electrochemical biosensor	0.05–20	0.04	[52]
Graphene/carbon nanosphere electrochemical immunosensor	0.05–15	0.02	[53]
ELISA (EnviroGard)	0.2–4.0	0.1	-
ELISA (EnviroLogix)	0.16–2.5	0.147	-
ELISA (Abraxis)	0.15–5.0	0.1	-
Aptatoxisensor	0.1–1.1	0.04	This study

DLR: Dynamic linear range; MALDI-TOF MS: Matrix-assisted laser desorption/ionization time-of-flight mass spectrometry; CLEIA: Chemiluminescence enzyme immunoassay.
